# Detection of Prion Protein Particles in Blood Plasma of Scrapie Infected Sheep

**DOI:** 10.1371/journal.pone.0036620

**Published:** 2012-05-02

**Authors:** Oliver Bannach, Eva Birkmann, Elke Reinartz, Karl-Erich Jaeger, Jan P. M. Langeveld, Robert G. Rohwer, Luisa Gregori, Linda A. Terry, Dieter Willbold, Detlev Riesner

**Affiliations:** 1 Institute of Physical Biology, Heinrich-Heine-University Düsseldorf, Düsseldorf, Germany; 2 Institute of Complex Systems (ICS-6), Research Center Jülich, Jülich, Germany; 3 Institute of Molecular Enzyme Technology, Heinrich-Heine-University Düsseldorf, Research Center Jülich, Jülich, Germany; 4 Central Veterinary Institute of Wageningen UR (CVI), Lelystad, The Netherlands; 5 VA Maryland Health Care System, Molecular Neurovirology Laboratory, Medical Research Service 151, VA Medical Center, Baltimore, Maryland, United States of America; 6 Animal Health and Veterinary Laboratories Agency, New Haw, Addlestone, Surrey, United Kingdom; 7 Department of Neurology, University of Maryland at Baltimore, Baltimore, Maryland, United States of America; Creighton University, United States of America

## Abstract

Prion diseases are transmissible neurodegenerative diseases affecting humans and animals. The agent of the disease is the prion consisting mainly, if not solely, of a misfolded and aggregated isoform of the host-encoded prion protein (PrP). Transmission of prions can occur naturally but also accidentally, e.g. by blood transfusion, which has raised serious concerns about blood product safety and emphasized the need for a reliable diagnostic test. In this report we present a method based on surface-FIDA (fluorescence intensity distribution analysis), that exploits the high state of molecular aggregation of PrP as an unequivocal diagnostic marker of the disease, and show that it can detect infection in blood. To prepare PrP aggregates from blood plasma we introduced a detergent and lipase treatment to separate PrP from blood lipophilic components. Prion protein aggregates were subsequently precipitated by phosphotungstic acid, immobilized on a glass surface by covalently bound capture antibodies, and finally labeled with fluorescent antibody probes. Individual PrP aggregates were visualized by laser scanning microscopy where signal intensity was proportional to aggregate size. After signal processing to remove the background from low fluorescence particles, fluorescence intensities of all remaining PrP particles were summed. We detected PrP aggregates in plasma samples from six out of ten scrapie-positive sheep with no false positives from uninfected sheep. Applying simultaneous intensity and size discrimination, ten out of ten samples from scrapie sheep could be differentiated from uninfected sheep. The implications for *ante mortem* diagnosis of prion diseases are discussed.

## Introduction

Prion diseases are fatal neurodegenerative diseases in humans and animals. Most prominent examples are scrapie in sheep, bovine spongiform encephalopathy (BSE) in cattle, chronic wasting disease in deer (CWD) and Creutzfeldt-Jakob disease (CJD) in humans. A characteristic feature of prion diseases is the accumulation of a pathological isoform of the host-encoded prion protein (PrP). Whereas the cellular isoform, PrP^C^, is soluble in mild detergents, the pathological isoform, PrP^Sc^, forms insoluble aggregates. While PrP^C^ is highly sensitive to complete digestion with proteinase K (PK), PrP^Sc^ is only N-terminally truncated leaving the C-terminal part (aminoacids 90–231) undigested with high retention of infectivity. Thus, PrP^Sc^ embodies both a PK-resistant and a PK-sensitive portion; both moieties form aggregates, and neither can be detected in uninfected animals or humans. According to the prion hypothesis proposed by Stanley Prusiner, PrP^Sc^ is, by itself, the agent of this class of transmissible diseases [1]. The prion hypothesis has now been strongly corroborated by recent demonstrations of infectivity in particles prepared *in vitro* from recombinant PrP, though the PrP conformation(s) bearing infectivity still has to be clarified [2]–[4].

As for any transmissible disease, sensitive and reliable diagnostic procedures are obvious prerequisites to the control of transmission. To control the BSE epidemic not only in Europe, but also in Japan and Canada, an effective strategy of active monitoring is being carried out through *post mortem* testing on cattle brain tissue. All of these tests are based on detection of the PK-resistant forms of PrP^Sc^ except for a single test that detects PrP^Sc^ aggregates captured by an aggregate-specific ligand without PK digestion [5]. The BSE epidemic is now largely contained. Approximately 200 cases of variant CJD (vCJD) have shown, however, that BSE can cross the species barrier to human. Unresolved problems include the lack of sensitive live tests, incomplete knowledge of sources and routes of exposure and transmission, and means to assess, monitor and manage the public health risks from infected blood.

Transmission via blood has been shown in experimental rodents like hamster [6], [7] as well as in species naturally susceptible to prion diseases like sheep and deer [8], [9]. Moreover, some cases of secondary variant Creutzfeldt Jakob disease (svCJD) have been reported that were caused by blood transfusion from presymptomatic vCJD patients [10], [11]. Transfusion transmission occurs despite the low concentration of prion infectivity in blood, ∼10 infectious doses/ml in clinically affected rodents, or 7 to 9 orders of magnitude less than the concentration in the brains of symptomatic mice or hamsters [7]. *Post mortem* tests on brain samples can be carried out with high sensitivity and reliability, whereas qualitatively similar tests based on body fluids of afflicted humans or animals have yet to be developed. Blood tests are, however, highly desired for pathogenesis studies, blood transfusion safety and CJD-therapy assessment.

In recent years significant progress has been made in the field of prion diagnostics with the development of prion seeded amplification technologies like protein misfolding cyclic amplification (PMCA, [12]), quaking induced conversion (QuIC, [13]), and amyloid seeding assay (ASA, [14]). QuIC was successfully applied to cerebrospinal fluid samples from sporadic CJD patients [15], [16] and rodent blood [17]. Using PMCA, it has been possible to detect PrP^Sc^ in blood from prion-infected hamsters, sheep and deer [18]–[23]. At present, however, PMCA is carried out reliably, i.e. without false positives, only in highly specialized laboratories. In another development, PrP^Sc^ was detected in the peripheral mononuclear blood cells (PBMC) of scrapie-afflicted sheep [24], and in blood samples of variant CJD cases by an improved immune detection method of surface-captured prions that did not require the use of *in vitro* amplification and protease digestion [25].

In this study we have investigated PrP aggregates, PK-resistant as well as PK-sensitive forms, in blood plasma of scrapie-infected sheep. We have adapted our previously developed fluorescence intensity distribution analysis (surface-FIDA) technique [26], [27] for analysis of blood samples of sheep. PrP aggregates are partially purified from blood plasma, captured on a surface by covalently bound antibodies and made visible by fluorophore-labeled detection antibodies. The fluorescence emitted in response to a scanning laser beam is transformed into an image of the PrP fluorescence intensities on the surface. Several features of the method, e.g. sample preparation, detection, and data processing, guarantee that PrP aggregates can be differentiated safely from PrP^C^. We show that PrP aggregates are detectable in blood of scrapie-infected sheep and that their presence indicates scrapie infection.

## Materials and Methods

### Ethics Statement

All animal work was conducted in compliance with relevant national guidelines. The sheep plasma reference pool was created from blood collected in compliance with Institutional Animal Care and Use Committee approved Protocol #2000-17 issued by the University of Idaho. Animal procedures for gain of individual blood samples were approved by the UK home office under the Animals (Scientific Procedures) Act 1986.

### Preparation of recombinant PrP aggregates

Bacterial expression and purification of recombinant (rec) ovine PrP25–233 carrying the VRQ allele was performed as described elsewhere [28]. Protein was stored in 10 mM sodium phosphate buffer (NaPi) pH 7.2, supplemented with 0.2% sodium dodecyl sulfate (SDS). Amorphous aggregates of recPrP were obtained by reducing the SDS concentration in the monomer solution by dilution of the recPrP stock solution to100 to 200 ng/µl PrP in 10 mM NaPi pH 7.2 followed by incubation at 37°C for 16 hours [29]. Aggregation was monitored by ultracentrifugation at 100,000 *g* for 1 h followed by dot blot analysis of pellet and supernatant.

### Preparation of blood plasma

Blood was obtained from clinically-affected sheep (VRQ homozygous) naturally infected with scrapie and control sheep as described by Terry et al. [24]. Analyses of reproducibility were performed on aliquots of a reference plasma (Rohwer Lab, Batch 101 Grade A hemoglobin OD575 nm = 0.047) pooled from fresh frozen plasma collected from 42 scrapie-infected North American sheep of various breeds and genotypes. Sheep symptomatic with scrapie were exsanguinated into CP2D via the jugular vein during euthanasia under supervision of Dr. Marie Bulgin, at the Caine Veterinary Teaching Center, 1020 E. Homedale Rd., Caldwell, ID. 83607. Frozen plasma from 42 sheep were thawed at≤four degrees centigrade, and after reserving one or more 50 ml aliquots of each plasma for future studies, the remainder was combined and thoroughly but gently mixed before re-aliquoting and refreezing at< = −80C for storage.

Prior to Surface-FIDA analysis, plasma samples were thawed slowly on ice and treated with Complete Protease Inhibitor Cocktail (Roche, Mannheim, Germany). Gross debris was removed by brief 500×g centrifugation. Samples were adjusted to 2% (w/v) n-lauroylsarcosin (sarkosyl, Fluka Analytical, Buchs, Switzerland) and incubated for 15 min at 37°C. Afterwards, lipids and lipid moieties were digested by adding 1 µl Phospholipase C from *Clostridium perfringens* (Sigma Aldrich, St. Louis, MO, USA), 1 µl Phospholipase A_2_ from porcine pancreas (Sigma Aldrich), and 1 µl *Candida antarctica* lipase B (CalB, Novozym 435, Novozymes, Denmark) per 100 µl of blood plasma. Lipatic digestion was conducted for 1 h at 37°C on a rocking platform. As a final step PrP aggregates were concentrated by precipitation with 2% (w/v) phosphotungstic acid (PTA, Sigma Aldrich). The mixture was incubated for 1 h at 37°C and centrifuged for 30 min at 14.000×g in a tabletop centrifuge. Pellets were resuspended using ultrasound (30 sec, 170 W in a sonication bath).

### Surface-FIDA assay

The microplates (SensoPlate Plus 384 well glass bottom microplates, Greiner Bio One, Kremsmünster, Austria) consist of a flat borosilicate glass bottom and a black polystyrene frame. They are suitable for fluorescence measurements on the surface. To bind the capture antibody covalently to the glass surface we used the heterobifunctional polyethylene glycol spacer (NHS-PEG-COOH, MW 3.400, Laysan Bio, Arab, AL, USA) according to an optimized protocol for single molecule applications [30]. The glass surface was cleaned consecutively with 100 µl of 5 M NaOH and with 100 µl of 1 M HCl per well. It was rinsed with water and ethanol and then dried in a nitrogen stream. The glass was next immersed for 1 h in 50 mM PEG-spacer dissolved in dimethyl sulfoxide containing 2% (v/v) triethylamine (Fluka, Basel, Switzerland). After activation of the carboxylic end group of the spacer by 50 mM 1-Ethyl-3-(3-dimethylaminopropyl)carbodiimid and 50 mM N-Hydroxysuccinimide for 30 min, wells were washed with 2-(N-morpholino)ethanesulfonic acid (MES) buffer, pH 5.0. To crosslink the capture antibody to the glass, 1 µg of mAB SAF-32 (SPI-Bio, Montigny le Bretonneux, France), was added and incubated for 1 h. Antibody mAB SAF-32 binds to the octarepeat region located in the N-terminus of PrP. Unbound antibody was removed by washing three times with phosphate buffered saline (PBS). Finally 3% (w/v) bovine serum albumin containing 30 mM triethylamine was added for 1 h in order to quench remaining activated carboxylic end groups of the PEG-spacer. Before addition of the target, the wells of the microplate were washed 4 times with PBST (PBS, 0.2% Tween20) and 3 times with PBS.

The resuspended PTA pellets from the plasma preparations were added to the wells and incubated at 4°C for 16 h (optionally for 1 h at room temperature); unbound protein was removed from the captured aggregates by washing with PBST and PBS. For fluorescence detection 20 µl of 2.5 ng/µl Alexa-488 conjugated detection antibody SAF-32 or Alexa-633 conjugated antibody L42 were applied to each well and incubated for 1 h at ambient temperature. Unbound detection antibody was removed followed by three washing steps with PBST and three washing steps with PBS. Analysis of the samples was performed on the same day.

The device employed for surface-FIDA is a customized fluorescence correlation spectrometer (FCS Olympus IX 50, Evotec, Hamburg Germany). For surface imaging the instrument was upgraded with a MIPSS module (Modular Imaging and Photon Statistics System, Ionovation, Osnabrück, Germany). The scanning unit includes a ±25 mrad tilt mirror installed on a 2D piezo element, which is capable of scanning an image in the confocal plane. The emission of the fluorescent antibody probes is collected by high-sensitivity avalanche photo diodes, suitable for single molecule spectroscopy. Intensity binary data are collected and finally transformed into 16 bit images in tagged image file format (TIFF).

Prior to scanning, the laser is focused on the glass surface. Typically, for each well four to nine images each of 200 µm×200 µm were recorded at a resolution of 330 to 500 nm per pixel. For data evaluation the open source ImageJ tool was used (available at http://rsb.info.nih.gov/ij; developed by Wayne Rasband, National Institutes of Health, Bethesda, MD).

### Background reduction

**Figure 1:**
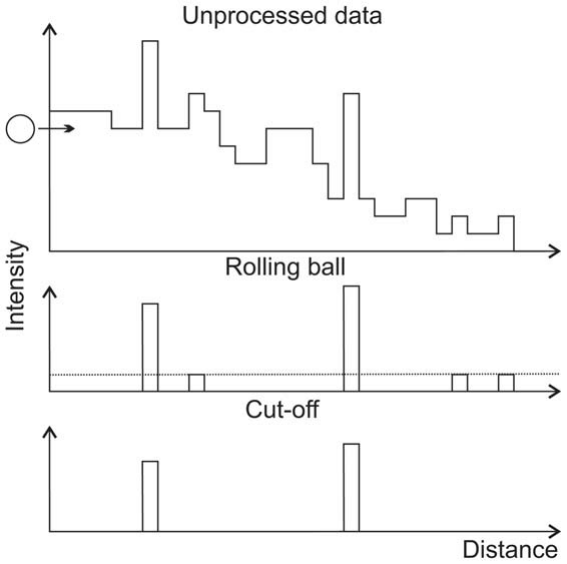
Schematic representation of the intensity distribution by an one-dimensional scan through the surface. A ‘rolling ball’ moves along (arrow) the underside of the intensity curve and thereby identifies the background, which is subsequently subtracted. Remaining peaks of low intensities are removed by an intensity cut-off (dashed line). In the algorithm, the ball moves along a three-dimensional landscape, identifying peaks with a two-dimensional bottom and the intensity as the third dimension.

The “subtract background" function in the ImageJ software employs the ‘rolling ball’ algorithm [31]. This algorithm not only compensates for uneven intensity distributions within an individual image, but is also capable of selecting for particular particle sizes. The image data can be visualized as a 2D surface with the pixel intensity as the third dimension. As shown schematically in Fig. 1 for a one-dimensional scan of the surface, a ball of a particular radius, selected as a parameter, is rolled along the underside of the surface. The ball can invade peaks of larger width but not those of smaller size. It thereby creates a local background distribution. Regions where the ball can go are subtracted from the image. As a general rule, the smaller the ball radius, the more background is removed. Narrow peaks of low intensities, which persist after ‘rolling ball’ processing, are subsequently removed by applying an intensity cut-off. For this purpose, a fixed value is subtracted from each pixel's intensity. Finally the remaining intensities of a sample are summed and displayed as a bar chart.

## Results

### Recovery of artificial PrP aggregates from plasma samples

**Figure 2:**
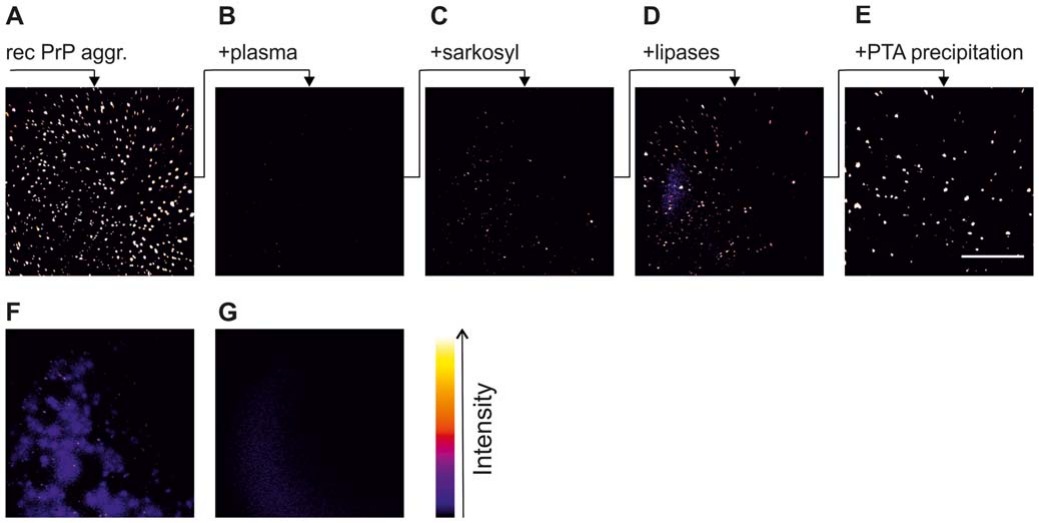
Detection of recPrP particles in the presence of blood plasma. To better resolve grayscales, images are colorized with ‘fire’ lookup table (cf. right bar). Each sample contained 50 ng recPrP aggregates in 100 µl sample volume. All samples were analyzed by surface-FIDA assay using SAF-32 as capture and detection antibody. One image corresponds to 1% of the total well bottom area. RecPrP aggregates (**A**) in PBS, (**B**) in ovine plasma, (**C**) in plasma and 2% sarkosyl, (**D**) in plasma after sarkosyl and lipase treatment, (**E**) in plasma after sarkosyl and lipase treatment and PTA-precipitation, (**F**) in plasma after PTA precipitation. (**G**) negative control: non-spiked plasma after sarkosyl, lipase and PTA steps.

Although surface-FIDA is sensitive enough to detect and quantify PrP aggregates at the single particle level, the sensitivity and reliability of the assay depends on the accessibility of PrP aggregate epitopes to the capture and detection antibodies. The conditions for preparation of plasma were optimized using aggregates of recombinant PrP. When 50 ng of preformed amorphous recPrP aggregates were added to 100 µl PBS buffer and the same amount was spiked into 100 µl EDTA plasma of sheep and analysed by surface-FIDA, very few PrP aggregates were detectable in PrP-spiked blood plasma compared to buffer (Figs. 2A, 2B). Safar and colleagues [32] reported that human prions are associated with low density lipoprotein (LDL and VLDL). Hence, we postulated that recPrP aggregates may also associate with these lipoproteins and other lipid particles when added to plasma thereby masking the epitopes available for capture and detection by antibodies. We therefore devised a strategy to expose the epitopes on recPrP aggregates by combining a detergent solubilization step with subsequent enzymatic hydrolysis of lipids. To disperse LDL-PrP complexes the spiked plasma samples were treated with 2% (w/v) n-lauroylsarcosin (sarkosyl) resulting in a moderate increase of detectable aggregates (barely visible in Fig. 2C). Detergent-solubilized samples were subsequently incubated with a mixture of phospholipase A, phospholipase C, and CalB lipase. This treatment significantly increased the amount of detectable aggregates (Fig. 2D). Finally, PrP aggregates were enriched by precipitation with PTA (Fig. 2E). Direct precipitation of spiked plasma without prior enzymatic treatment resulted in formless, diffuse signals (Fig. 2F), whereas lipolyzed plasma without PrP yielded very low background (Fig. 2G).

### Detection and quantitation of authentic PrP aggregates in natural blood samples

Once conditions for aggregate detection had been established, plasma pools prepared from uninfected and scrapie-affected sheep were assayed. In order to assess the reproducibility of the surface FIDA assay, we analyzed replicate samples from these plasma pools. Sample preparation was conducted independently by two experimenters to estimate operator error. For each sample, the PTA pellet was resuspended and then divided equally between two wells. After background reduction by intensity cut-off application, the mean of the signals from each pair of wells was used for comparisons between samples.

Preparation and measurement of the samples were performed over several days. Because factors like daily device calibration, brightness of fluorescent antibodies, variations in laser power, etc. have large impacts on absolute intensities, the fluorescence signals were normalized. Normalization provides a more easily interpretable discrimination between negative and positive samples. Normalization was performed by subtracting intensities of the negative control samples that were processed with the positive samples from the intensities of the positive samples (I_pos_−I_neg_). This difference was then divided by the intensity of the positive sample (I_pos_−I_neg_)/I_pos_. A value of 1, achievable only if the background is zero, reflects the maximum possible discrimination from background, a value close or below zero means no differentiation.

**Figure 3:**
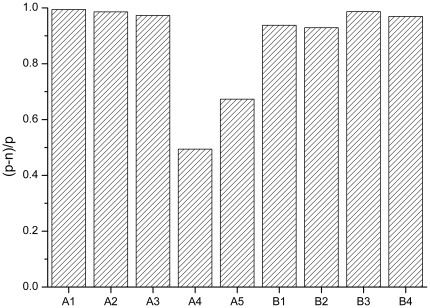
Reproducibility of plasma analyses. Replicate samples of a plasma pool prepared from sheep symptomatic for scrapie and from a pool prepared from uninfected control sheep were processed independently by two experimenters (A and B). Discriminability of positive and negative samples is expressed as fluorescence intensity (positive, p) minus intensity (negative, n) divided by intensity (positive, p). MAb SAF-32 was used as capture, mAb L42 as probe.

Results of this evaluation are depicted in Fig. 3. In the majority of the measurements the positive samples yielded more than a tenfold higher fluorescence intensity compared to the negative sample (discriminability >0.9). In only two out nine measurements was discriminability lower, 0.49 and 0.67. Since the samples were identical, these differences are apparently due to procedural variability which should be amenable to reduction. Even for these samples the discrimination was unambiguous i.e. the fluorescence from the positive sample was still higher than background by a factor of 2 and 3, respectively. Taken together, surface-FIDA is able to discriminate positive from negative plasma samples in a reproducible manner.

### Detection of PrP aggregates in individual blood samples

**Figure 4:**
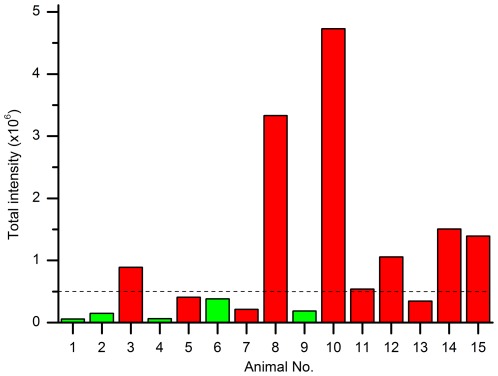
Detection of PrP particles in blood plasma of scrapie-infected sheep in a blinded study. PrP aggregates from 15 plasma samples were prepared, applied to surface-FIDA using mAb SAF-32 as capture and detection probe and evaluated using a rolling ball radius of 10 px. and intensity cut-off 200. After decoding samples were assigned as scrapie-positive (red) or uninfected controls (green).

We prepared and analyzed blood plasma from individual scrapie-infected sheep and healthy control sheep as described above. Samples were blinded before preparation and data evaluation. Images were subjected to background reduction (‘rolling ball’ radius = 10 px., cut-off = 200) as described in the method section, and the remaining intensities were summed and plotted as single values in Figure 4. A threshold value that clearly separated stronger from weaker signals was assigned. Samples with intensities well above the indicated threshold value (dashed line, Fig. 4) were classified as positive. After unblinding the samples by our colleagues at the AHVLA (Weybridge), we observed that 6 out of 10 scrapie-positive clinical animals had been identified as positive without any false-positives.

### Optimization of background removal

**Figure 5:**
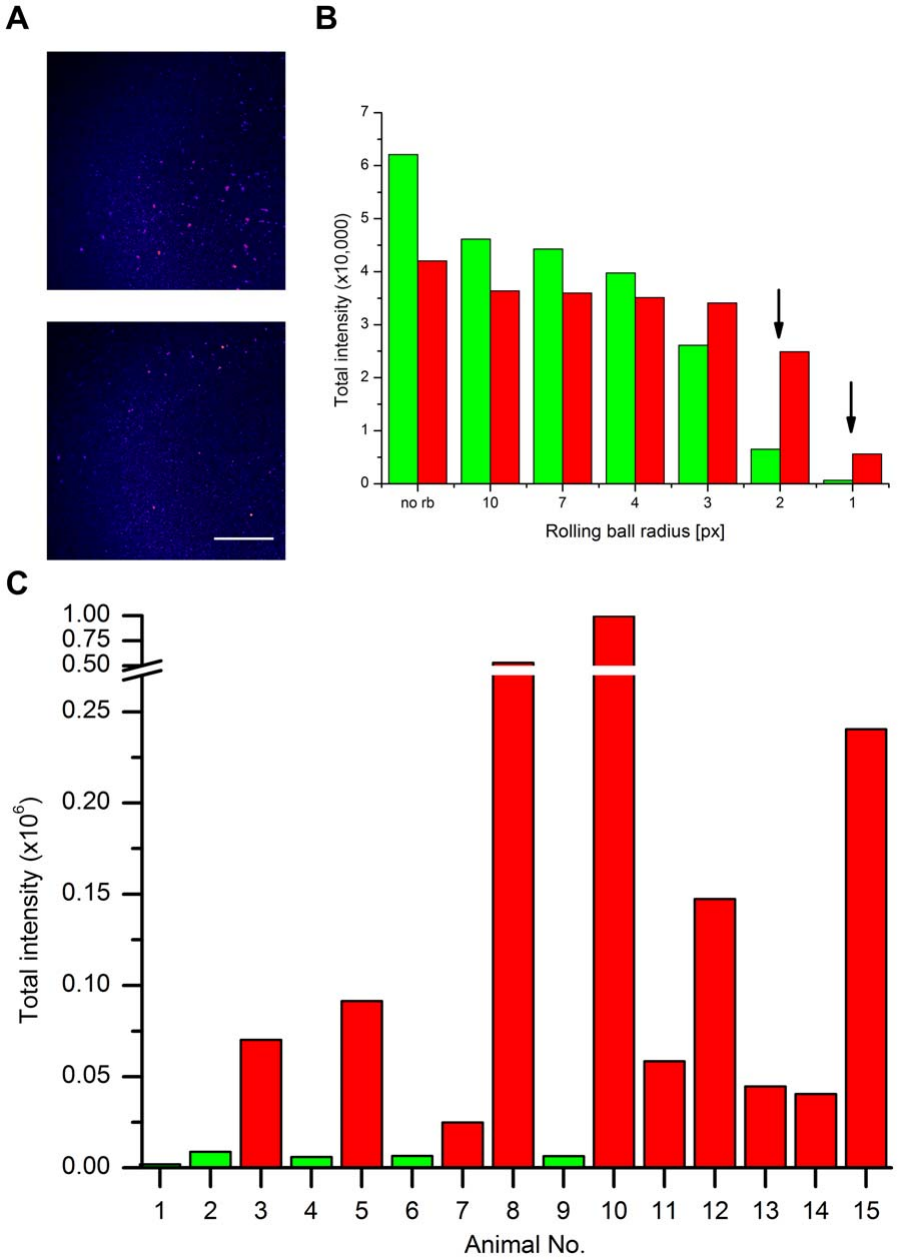
Optimized image processing and evaluation. (**A**) Image raw data of control sample no. 6 (top) and a scrapie-positive sample no. 7 (bottom). Depicted is one representative out of nine images taken for each sample. Scale bar = 50 µm. (**B**) Optimization of ‘rolling ball’ radius. After applying rolling ball background subtraction an intensity cutoff (500) was applied. Smaller radii of 1 and 2 pixels (arrows) allow for differentiation of the positive and the control sample (negative). **(C)** Optimized background removal parameters were applied to the complete panel of samples.

The cutoff and threshold values assigned to the blinded samples were by necessity somewhat arbitrary. Once sample identity had been disclosed, background reduction parameters could be optimized in order to maximize signal-to-noise from the FIDA analysis. Optimizations were performed on two exceptionally critical samples: Negative sample no. 6 yielded the highest intensity among all controls, while positive sample no. 7 showed the lowest signal among all clinical samples (Fig. 4). Both samples show an uneven background, the control even higher than the positive sample. In the control fairly large particles are visible, in the positive sample significantly smaller particles (Fig. 5A). The radius of the ‘rolling ball’ was varied from 1 to 10 px, followed by an intensity cut-off of 500 (Fig. 5B). Remarkably, differentiation of the negative and the positive sample was achieved at radii of 1 and 2 px (corresponding to 500 and 1000 nm). Moreover with these parameters all ten samples from scrapie-affected sheep were differentiated from uninfected control sheep (Fig. 5C). Therefore, it is the small but highly intense particles that best discriminate scrapie infected from uninfected plasma and must also therefore be disease-specific.

## Discussion

### Methodological aspects

The method of surface-FIDA was developed earlier [26] and in the present work has been applied to analyze authentic PrP aggregates in blood of sheep. It was the intention of this study, first to detect and characterize PrP particles in blood and second to determine if surface-FIDA would be appropriate for a live test for scrapie infection in individual sheep. The sensitivity was expected to be higher compared to ELISA, even though in both methods the fluorescent analyte is bound to a surface. However in surface-FIDA individual particles are discriminated from their neighboring background, whereas in ELISA an integrated signal of the whole surface is obtained and compared to the integrated signal from a control sample. Recently, Terry et al. [24] reported an improved ELISA test, in which the authors detected PrP^Sc^ in PBMCs from 55% of scrapie-infected sheep in a blind panel. The blood plasma samples tested in our study were derived from the blood panel used to produce the corresponding cellular samples tested by Terry and colleagues. A comparison of the results showed that both assays had a total sensitivity of 55–60%, and both detected the same 60% of positive cases. It should be noted, however, that infectivity in the PBMC fraction is about one order of magnitude higher compared to blood plasma [6], [7], and prions were not detected in plasma by the ELISA method reported by Terry et al.

In an earlier study, we described the detection of PrP aggregates with high sensitivity in brain homogenate of BSE cattle, and in a small number of cerebrospinal fluid samples from BSE cattle [26]. According to the literature, infectivity in blood - even in symptomatic experimental hamsters - is as low as 10 infectious units per ml [33], [34]. In BSE-afflicted cattle infectivity is absent from the lymphatic system and has never been reported in blood [35], [36]. However, seeding activity was demonstrated in a small number of BSE serum samples [37]. Considerable effort was spent not only in improving the sensitivity of the assay but also in optimizing the preparation of PrP aggregates from blood plasma. Though it is not certain that the PrP aggregates we analyzed are indeed the carriers of infectivity in blood, they are a consistent marker of infection. The direct determination of infectivity in these PrP aggregates from blood remains to be established.

Although PrP particle number was expected to be very small in blood plasma, it is clear from our data that PrP aggregates are not only associated with cells, like peripheral mononuclear blood cells prepared from buffy coat, but are also present in plasma. A PK-digestion step was avoided because it had been shown earlier that some portion of PrP aggregates is PK-sensitive. Spiking of blood plasma with PrP^Sc^ from brain for method development was not successful in our hands, because the properties of PrP^Sc^-samples from brain proved to be very different from those of the genuine PrP aggregates in blood. Recombinant polymorphous PrP aggregates behaved more suitably as spiking material because they remained stable in plasma and allowed us to develop procedures to overcome epitope masking for antibody binding. We assume that PrP aggregates are complexed in plasma by lipoproteins and lipids as described by Safar and colleagues [32] and the epitopes were unmasked successfully by treatment with a mild detergent and a lipase cocktail. Finally, precipitation with PTA under optimized conditions was effective in concentrating the few PrP aggregates in blood plasma.

MAb SAF-32 was used both for capture and detection in the serial measurements (Fig. 4), although mAb L42 could also be successfully applied as probe (Fig. 3). SAF-32 has the advantage of more epitopes in the octarepeat region, resulting in higher sensitivity. A disadvantage, however, could be that SAF-32 epitopes are not present anymore in PrP27–30, i.e. after truncation at amino acid 89/90. In order to increase the sensitivity of PrP aggregate analysis, background signals had to be suppressed. The antibodies used for capture as well as detection may bind non-specifically to cell fractions and other non-PrP compounds in the sample. We tried to solve this problem by using two different detection probes simultaneously in dual-wavelength mode differentiating specific and background signals by co-localization. Unfortunately, non-specific antibody binding occurred with different antibodies in a similar manner, possibly mediated by the conserved region. Thus, background could not be suppressed further by means of dual-color measurements. Use of a different set of antibodies might overcome this problem. Moreover, additional probes like amyloid binding dyes or antibodies directed against PrP^Sc^ [38], [39] might further increase specificity.

### PrP aggregates in plasma of infected sheep

PrP aggregates have been detected unequivocally in blood plasma of scrapie-infected sheep. The sum of the fluorescence intensities of all particles in one well is more than an order of magnitude over background in some animals; in other animals it is, however, close to the corresponding signal from non-infected animals. All blinded samples which were assigned as positive, were indeed positive after unblinding; no false-positives were among the controls. The variability in the signals from scrapie positive animals might be a consequence of the variable extent of infection or dissemination via the blood plasma. However, some variability from our preparation and measurements cannot be excluded. The handling of small, nearly invisible pellets from the PTA precipition was critical and an erroneous result could be due to a loss of otherwise detectable PrP aggregates at that step. A detailed analysis of experimental errors was carried out with replicate determinations on standardized plasma pools prepared from scrapie and control sheep (cf. Fig. 4).

When we analyzed the blinded series of samples, we selected an assay threshold which clearly separated a number of larger signals from lesser signals regarded as background (Fig. 5A). After decoding we had identified 60 to 70% of the positive samples with no false positive samples. Once the infection status of all samples was known we found that a rolling ball correction with a radius of 2 pixel and cut-off of 500 intensity units could discriminate 100% of the positive samples from the negative samples (cf. Fig. 5B). By using a 2 pixel background selection, only particles with a radius smaller than 2 pixel, i.e. 660 nm were counted as PrP aggregates. By specifying a high intensity cut-off of 500 fluorescence units only bright particles were counted. We cannot exclude the existence of larger PrP aggregates in blood, but they could not be differentiated from the background. The best correlation between disease and PrP aggregates in plasma was clearly achieved by filtering small and bright aggregates.

At least two factors contribute to the size distribution of the PrP aggregates: the genuine *in vivo* size distribution and the break-down of larger aggregates by the ultrasonication step during the preparation from plasma.

We estimate a particle size for the aggregates selected for discrimination in Figure 5B of about a micrometer, after subtracting 50 to 100 nm for the size of the fluorescent antibody label. Particle sizes in the literature were derived from nanofiltration studies investigating the removal of infectivity from blood or plasma. Summarizing the studies applying different filters, preparation procedures and solution conditions lower limits of the particle sizes between 15 and 200 nm were estimated [40]–[47].Thus the lower limit found in filtration experiments and the upper limit reported here do not contradict each other. Sonication can affect PrP aggregate size, but does not break down the aggregates below the size of infectious particles. A rough estimation of the number of PrP molecules in PrP aggregates as observed in plasma leads to 10^5^ to 10^6^ molecules, which corresponds to an earlier estimation of PrP molecules per infectious unit in brain [48]. While our estimates of particle size are derived from natural PrP aggregates in plasma, it must be pointed out that all of the precedent filtration studies on plasma have, by necessity, employed brain or spleen derived tissue homogenates as a source of PrP^Sc^ and infectivity. This is because there has not been an assay sensitive enough to detect disease-specific PrP in blood. This is the first direct measurement of the size of endogenous disease-specific PrP aggregates in plasma. It should be noted that particular, detergent-including preparation of infectious particles from brain led to determination of smaller infectious units [49].

### Surface-FIDA for live test

This study has demonstrated that disease-specific PrP aggregates can be detected in blood plasma. As a consequence, the method employed, surface-FIDA, could appropriately be used to test living sheep for scrapie infection. So far we have only tested symptomatic sheep. With a signal over background ratio of over ten for symptomatic animals, it is likely that the assay can also be extended to sheep in the preclinical stage. Refinements of concentration, detection modalities, and instrumentation may further extend the sensitivity. A self-modified fluorescence correlation spectrometer was used for the measurements presented here. In the meantime autofocussing laser scanning microscopes with a high degree of automation have appeared on the market which should improve the quality of the analysis. Labeling with two different antibodies did not significantly improve the analysis in the present work but might be applied more successfully in the future using different antibodies e.g. one sequence specific and one structure specific antibody, or by using fibril-specific dyes like Thioflavin T as a second probe. QuIC and PMCA are probably the more sensitive tests. Surface-FIDA has the potential to be much faster than PMCA and may be more amenable to high throughput. Because amplification technologies and surface-FIDA rely on different properties of the infectivity, i.e. seeding activity versus presence of PrP particles, the two assays complement each other as valuable cross-confirmatory tests. Surface-FIDA might be used in combination with QuIC or PMCA to further increase sensitivity or speed of that assay.
